# Supplementary material for: Reprogramming‐derived gene cocktail increases cardiomyocyte proliferation for heart regeneration
Source: EMBO Mol Med. 2016 Dec 23;9(2):251–64. doi: 10.15252/emmm.201606558 (PMC5286362; doi:10.15252/emmm.201606558)
Supplement: Supplementary file 1 — Appendix [file EMMM-9-251-s001.pdf]

Appendix

Reprogramming-derived Gene Cocktail Increases Cardiomyocyte Proliferation for Heart Regeneration

Yuan-Yuan Cheng, Yu-Ting Yan, David J. Lundy, Annie H.A. Lo,

Yu-Ping Wang, Shu-Chian Ruan, Po-Ju Lin & Patrick C.H. Hsieh

Four appendix tables

Six appendix figures

Appendix Tables

Table S1. List of genes from microarray showing > 2-fold up- or down-regulation at day 2 of CM reprogramming

**Up-regulation**					
**Probe Name**	**Gene Symbol**	**Fold Change**	**Probe Name**	**Gene Symbol**	**Fold Change**
A_55_P1983858	Scel	45.64251	A_52_P232637	Dhh	4.7735643
A_51_P468260	S100b	30.435705	A_55_P2324976	5033406O09Rik	4.7283297
A_52_P449871	Id4	22.281864	A_66_P129622	Phldb3	4.687701
A_52_P65237	Zbtb7c	13.476518	A_55_P2072561	Gm3065	4.5743523
A_52_P734742	Lrrc73	9.040887	A_51_P346165	Agpat4	4.557826
A_55_P2128153	Col6a1	8.170807	A_55_P2078365	Rgs12	4.5333815
A_55_P1960157	Bcat1	7.06457	A_55_P2060243	Itgbl1	4.4744444
A_55_P2394308	Fst	6.9435678	A_51_P342871	S100a10	4.420988
A_55_P2435563	Etl4	6.8120604	A_51_P446510	Emp3	4.39257
A_55_P2052563	Id1	6.740239	A_55_P2023188	Lsm2	4.327197
A_52_P60353	Greb1l	6.6704416	A_51_P386539	Rnf125	4.3269434
A_51_P109258	Cys1	6.6209254	A_55_P2060592	Hoxa1	4.1997914
A_55_P1993723	Acsbg2	6.5329037	A_51_P273556	Fam83d	4.17568
A_55_P2044143	Loxl4	6.458679	A_55_P2088615	Fbln5	4.146144
A_52_P281145	Kank4	6.4442263	A_55_P2176792	Sh3gl3	4.115314
A_51_P497724	Apol7a	6.3823647	A_55_P1954221	Emp1	4.046907
A_52_P381484	Spon2	6.2921624	A_55_P1992572	Celf4	4.0398936
A_55_P1962224	Afap1l2	6.124147	A_55_P1978962	Slc9a3r2	4.038525
A_52_P216672	Klk8	6.088077	A_66_P103654	Asxl3	4.0326796
A_55_P2059765	Foxf1	6.0161486	A_66_P109460	Mtfr2	3.9742641
A_52_P78373	Sorbs3	6.0027595	A_66_P136228	Rai14	3.964933
A_55_P2116180	Mrgprf	5.9930716	A_51_P453475	Slc1a5	3.9449556
A_51_P481159	Cbr3	5.9385004	A_55_P1996946	Cdc20	3.9338164
A_55_P1982404	Gpm6b	5.934873	A_51_P519008	Mkx	3.8981695
A_52_P187940	Lfng	5.7675996	A_55_P1997141	Mybl2	3.890319
A_52_P479262	Col6a3	5.6138806	A_51_P284665	Plcb1	3.884924
A_66_P107231	Loxl4	5.5048423	A_51_P427516	Thsd1	3.868489

A_55_P2282969	Gm2a	5.4894214	A_51_P344566	Plk1	3.864454
A_55_P2043627	Fam89a	5.430696	A_55_P2054663	Cox4i2	3.8643885
A_55_P1960238	Slc2a6	5.430179	A_55_P2068892	Il6ra	3.8381197
A_55_P1978927	Srgap1	5.2673154	A_55_P2004248	Apcdd1	3.8375382
A_51_P245368	Abcb1b	5.0189505	A_51_P362661	Spin4	3.8306196
A_55_P2079669	Bcat1	4.99613	A_52_P110534	Ttk	3.8258631
A_55_P2041828	Tubb3	4.9266286	A_52_P162099	Ckap2	3.8220913
A_55_P2091196	Hrct1	4.8966403	A_55_P2089035	Pole	3.820475
A_52_P106620	Tnfrsf11b	4.856102	A_55_P2025033	Cep55	3.8159385
A_52_P190647	Mxd3	3.8075	A_51_P255456	Cyp1b1	3.4338624
A_66_P134542	Anln	3.7966588	A_55_P2050439	Dlgap5	3.4318545
A_55_P2065671	Ccnb1	3.7957191	A_52_P411003	Dlgap5	3.4307065
A_55_P2136318	Slc1a5	3.794008	A_55_P1966332	Plac9a	3.4234562
A_52_P520466	Kif18b	3.7776713	A_55_P2100209	Kif2c	3.413538
A_55_P2090060	Mab21l3	3.7619236	A_55_P2022211	Plxdc2	3.4097457
A_51_P125205	Aqp1	3.7459762	A_55_P2061739	Tmsb4x	3.4020982
A_51_P270355	Dclk1	3.7252653	A_55_P2076048	Nuf2	3.4011672
A_51_P363749	Irf6	3.7250223	A_55_P1991605	Aplp1	3.3956993
A_52_P148212	Mis18bp1	3.655101	A_51_P515120	Hs3st3a1	3.3558095
A_55_P2023692	Neurl1a	3.6530645	A_51_P275697	Fam101b	3.3437078
A_51_P324287	Kif23	3.6165519	A_55_P2172201	Zfp57	3.3272264
A_55_P1966327	Plac9a	3.6114252	A_52_P35057	Kctd17	3.3261037
A_55_P2128668	Ccnb1	3.608869	A_51_P233928	B4galnt1	3.3256085
A_55_P2111478	Polq	3.603814	A_51_P288876	Tmem45a	3.3254223
A_51_P481920	Ccna2	3.5933723	A_55_P2139889	Zfp783	3.3180504
A_55_P1996941	Ube2c	3.5617204	A_52_P628067	Cdca3	3.312787
A_51_P253803	Mki67	3.5542693	A_55_P1988228	Aspm	3.3036397
A_52_P588881	Iqgap3	3.5460815	A_51_P481398	Kif11	3.2819011
A_66_P114695	Cdc25c	3.5441322	A_55_P2158011	Knstrn	3.2691545
A_52_P515769	Pcdh12	3.5343127	A_51_P501018	Nek2	3.2683141
A_55_P1952256	Ccnb1	3.5285118	A_51_P289889	Npnt	3.2646446
A_51_P451151	Ube2c	3.5246308	A_51_P164014	Cenpe	3.2354968
A_55_P1983450	S100a4	3.5200028	A_52_P251366	Neil3	3.2346928
A_51_P381260	Fxyd5	3.5123565	A_51_P279712	Rell1	3.2332022

A_51_P303749	Depdc1b	3.5091054	A_55_P1994997	Arhgap11a	3.2256691
A_55_P2173313	Plcb1	3.5016758	A_55_P2164957	Slc9a3r2	3.2222402
A_55_P2016618	Neurl1a	3.49488	A_51_P320022	Atp10a	3.2138982
A_51_P140641	Eva1a	3.482435	A_55_P2059010	Rbp1	3.2074208
A_51_P240453	Nusap1	3.4736712	A_52_P399584	Ckap2l	3.2073534
A_55_P2073377	Mki67	3.4709878	A_66_P125209	Kif2c	3.1988144
A_55_P2096947	Ereg	3.462979	A_55_P1983448	S100a4	3.194373
A_55_P2114201	G2e3	3.4441366	A_55_P1985433	Nrg1	3.192924
A_55_P2012389	Sfxn3	3.4359188	A_55_P1983769	Birc5	3.1925611
A_51_P326994	Calhm2	3.4341128	A_52_P27020	Sgol2	3.187126
A_55_P2181928	Slc45a3	3.1808927	A_55_P2025790	Fanci	3.1838439
A_52_P658437	Espl1	3.168732	A_52_P633714	Troap	2.9917948
A_51_P155142	Cdca8	3.1658123	A_52_P30989	Cdkn3	2.987538
A_52_P139650	Ska1	3.159886	A_51_P369200	Tpx2	2.9870327
A_51_P254805	Kif4	3.1572728	A_55_P1980631	Aurkb	2.9868243
A_51_P396983	Ssu2	3.1478918	A_51_P481693	Ero1l	2.9864867
A_55_P1988083	Prc1	3.1477556	A_55_P1983768	Birc5	2.9853733
A_55_P1983773	Birc5	3.1413915	A_51_P457196	Sfrp4	2.9806476
A_55_P2108768	Col6a2	3.1360626	A_52_P556099	Oip5	2.9777246
A_55_P1999633	Cit	3.1314445	A_55_P2105152	Trim59	2.9771929
A_55_P2037439	Depdc1a	3.13088	A_51_P139651	Nos3	2.9695275
A_51_P418116	Tmem119	3.1297226	A_52_P52849	Cpxm2	2.9664185
A_55_P1995007	Arhgap11a	3.1269655	A_55_P2000743	Kirrel	2.949359
A_52_P5891	Fam101b	3.1164098	A_51_P455897	Fam64a	2.94258
A_55_P2167461	Homer3	3.100387	A_55_P2095271	Pkn3	2.9319787
A_55_P2015032	Kazald1	3.0879328	A_55_P1991718	Hmmr	2.930425
A_55_P1968928	Def6	3.0839853	A_52_P20906	Twist1	2.9292948
A_55_P2056654	Kif22	3.0838487	A_51_P133137	Kif20a	2.9271085
A_55_P2119985	Baiap2	3.0773764	A_51_P519791	Ska3	2.9265118
A_55_P1982049	Icam2	3.0695086	A_55_P2005838	Ticrr	2.9240434
A_51_P492830	Cenph	3.0630379	A_51_P117739	Figf	2.9030569
A_55_P1958537	Map6	3.060607	A_55_P2170681	Cdca8	2.902731
A_55_P2176145	Rgs17	3.0523524	A_51_P195153	Gtse1	2.9025357
A_55_P1989321	Sat1	3.0402265	A_52_P460537	Lpar2	2.9015813

A_51_P123405	Bub1	3.0397656	A_66_P114627	S100a16	2.9014978
A_51_P457528	Ccnb2	3.0338523	A_66_P114451	Gpr126	2.899111
A_51_P513530	Spag5	3.0261045	A_52_P452569	Kirrel	2.897702
A_51_P412914	Efs	3.0213552	A_52_P420466	Hist1h2ab	2.897575
A_55_P2149951	Prx	3.0189447	A_55_P2166501	Cd44	2.8958702
A_55_P1976127	Ect2	3.01776	A_51_P125135	Cdca5	2.8920653
A_55_P1976584	Tmod2	3.0152657	A_52_P229052	Tmeff2	2.8911204
A_51_P211765	Rin1	3.0149472	A_55_P2007273	Pole	2.8881862
A_51_P302139	Mastl	3.0054991	A_55_P2059931	Prom1	2.8828073
A_52_P211223	Cdca2	3.0046873	A_55_P1984307	Gpr116	2.869508
A_55_P2151056	Rarg	3.0039883	A_51_P490509	Bub1b	2.863056
A_51_P172231	Gsdmd	2.9942446	A_52_P348250	Klhl29	2.8597836
A_52_P476431	Evc	2.8566508	A_51_P100174	Mns1	2.8575475
A_51_P137111	Chek2	2.8500872	A_55_P2024993	Hoxb4	2.7260926
A_66_P138137	Pnp	2.8471012	A_51_P105709	Trip13	2.723325
A_55_P2030938	Trim59	2.8466938	A_51_P108252	Gpsm2	2.7196949
A_55_P1972720	Pmm1	2.8463938	A_55_P1956862	Egfr	2.7190154
A_55_P2197914	Srgap1	2.8404524	A_52_P125467	Reep2	2.7166452
A_55_P2017362	BC068157	2.836458	A_55_P2189893	A630073K07Rik	2.7166097
A_55_P2013336	Melk	2.8341742	A_55_P2000280	Mthfd1l	2.7097523
A_55_P2266880	Kirrel	2.8330197	A_51_P513032	Trps1	2.7034926
A_51_P204402	Shcbp1	2.8141005	A_51_P429276	Tmod3	2.6951137
A_66_P133404	2810417H13Rik	2.8033478	A_55_P2010116	Rab27b	2.6950455
A_51_P487999	Sgol1	2.8031306	A_55_P2064043	Cd44	2.6945493
A_52_P206613	Adss	2.8008156	A_55_P2004801	Tacc3	2.6939092
A_55_P2171493	BC030867	2.7987585	A_55_P2014665	Racgap1	2.6892688
A_66_P125035	Duxbl2	2.7983406	A_55_P2038358	Acot1	2.6881132
A_55_P2037454	Etv5	2.7949345	A_55_P2015485	Ccdc19	2.6833737
A_51_P491987	Ripk3	2.793135	A_51_P520849	Sfrp2	2.682533
A_51_P172085	Arhgdig	2.792364	A_51_P270949	Hist1h1b	2.6749337
A_51_P151586	Gsg2	2.790252	A_51_P288916	Tmtc2	2.6694012
A_55_P2064984	Cenpi	2.781655	A_55_P1990341	Ncapg	2.6652567
A_55_P1987028	Kifc5b	2.7790415	A_55_P2077558	Sod3	2.6626797
A_52_P502771	Rad54b	2.778432	A_51_P408071	Kntc1	2.660122

A_51_P123134	Ercc6l	2.7773025	A_55_P1959393	Hhat	2.6530623
A_55_P1995205	Top2a	2.772372	A_51_P220135	Nfatc1	2.6528249
A_55_P2040743	Zwilch	2.769303	A_55_P2109717	Kif20b	2.634895
A_51_P463087	Cenpm	2.767622	A_51_P467224	Fbn1	2.631515
A_66_P130541	Traip	2.764734	A_55_P1972719	Pmm1	2.631147
A_55_P2000943	Nrip3	2.7615218	A_55_P2103796	Arhgap11a	2.6174133
A_55_P2012171	Spata6	2.7538335	A_55_P2099961	Hist1h2ag	2.6141691
A_55_P2204804	Dclk1	2.75351	A_55_P1965154	Spc25	2.6123686
A_65_P05396	Rad18	2.7470057	A_51_P190111	Mcm5	2.6037714
A_51_P390967	Slc43a3	2.7462025	A_55_P2127702	Racgap1	2.602209
A_52_P167278	Mthfd1l	2.7444296	A_51_P293688	Rab32	2.6018975
A_52_P28806	Foxm1	2.7397501	A_52_P350537	Mtmr11	2.5924518
A_52_P518997	Epha2	2.7292318	A_55_P1967291	Ncaph	2.5863605
A_52_P114905	Ifitm10	2.7282917	A_51_P487813	Lxn	2.5861104
A_52_P201206	Scrn1	2.574254	A_55_P2010622	Sec16b	2.5751579
A_55_P2034027	Nos3	2.574151	A_55_P2038247	Tyro3	2.457083
A_55_P2035286	Uhrf1	2.5687377	A_55_P1989981	Fam129b	2.4498599
A_52_P269942	Cpt1c	2.5683703	A_55_P2150343	Fam38a	2.4470646
A_55_P2121456	Fam43a	2.5577354	A_52_P191567	Plcl1	2.445575
A_55_P2110713	Anxa2	2.5563364	A_55_P2184449	Gm10387	2.4449475
A_51_P148105	Rad51	2.5527055	A_55_P1955279	Specc1	2.4449325
A_51_P314907	Dbf4	2.5487869	A_55_P2121521	Hist1h3i	2.4449096
A_55_P2147083	Tnfaip6	2.546718	A_55_P2056473	Spc24	2.4433556
A_55_P1969341	Brip1	2.5438125	A_55_P2048588	Cdk1	2.4433448
A_52_P201800	Nanos1	2.5431135	A_55_P2018904	Gm5465	2.4429693
A_55_P1975695	Snx18	2.5315144	A_51_P254045	Traip	2.4403632
A_55_P2152427	Zwilch	2.5280752	A_55_P2164534	Dtl	2.4399781
A_55_P2057686	Cdc42ep5	2.5255249	A_66_P120603	Trps1	2.4393723
A_51_P394802	Fam111a	2.523401	A_55_P1962747	H2-Ab1	2.438183
A_52_P104824	Diap3	2.519928	A_55_P2097553	C77080	2.423738
A_55_P2186648	Bard1	2.517406	A_55_P1987715	Epb4.1l2	2.4219706
A_55_P1982818	Odc1	2.5166903	A_66_P120074	Uhrf1	2.4176254
A_66_P131979	Cdc6	2.5136158	A_65_P20641	Fads2	2.4138472
A_52_P529570	Nsl1	2.5123994	A_51_P393958	Fbxo5	2.410691

A_52_P498208	Hist1h2ak	2.5107698	A_52_P527106	Arhgap12	2.406184
A_52_P393314	P2rx7	2.5015006	A_52_P462472	C77370	2.4034355
A_55_P1987331	Prl3d1	2.500798	A_55_P2030721	Ankle1	2.400851
A_51_P196973	Chaf1a	2.4958587	A_51_P424810	Ncapg2	2.3999689
A_55_P1991688	Rad51ap1	2.495593	A_55_P2005585	Trps1	2.3918388
A_55_P2069221	Prr11	2.4890509	A_55_P2004881	Ldlrad4	2.3908496
A_51_P240614	Tm4sf1	2.4834535	A_66_P118513	11-Sep	2.388504
A_51_P204442	Phf19	2.4739256	A_55_P2094524	Gm14005	2.3874974
A_55_P2062543	Kifc1	2.469086	A_55_P2041961	Bmp2	2.3869092
A_51_P317031	Ccdc109b	2.46812	A_55_P1981241	Grasp	2.3807874
A_55_P2098200	Tpbg	2.4677062	A_51_P332355	Kif18a	2.3800998
A_55_P2101757	Dscc1	2.4662392	A_55_P2085412	Ankle1	2.3758974
A_55_P2084631	Hist1h2an	2.463547	A_52_P380369	Phf11d	2.3752544
A_55_P2007389	Fam189b	2.4622793	A_51_P367310	Chaf1b	2.3751657
A_55_P2000778	Kif7	2.4617934	A_55_P2036883	Hist2h2bb	2.3737593
A_55_P2140031	Fam129b	2.4605255	A_55_P1978201	Incenp	2.3720763
A_66_P108143	Fbxw10	2.3673067	A_52_P634090	Jag1	2.2472148
A_51_P453043	Aacs	2.364335	A_55_P1989296	Tnxb	2.238779
A_55_P2048119	Slc29a4	2.3622744	A_51_P306218	Xkr5	2.236555
A_51_P299216	Wdr90	2.3594036	A_51_P246339	Rfc5	2.235493
A_52_P457967	Trerf1	2.3496401	A_66_P134481	Skp2	2.2353992
A_55_P2041070	Tle3	2.347125	A_51_P128876	Ifitm3	2.2333114
A_55_P2019463	Tnks1bp1	2.3452613	A_55_P1961730	Kcnt2	2.2300813
A_51_P432794	Fam115c	2.344881	A_55_P2012166	Spata6	2.2292001
A_52_P127682	Dagla	2.3433754	A_55_P2154252	Gfpt2	2.226789
A_55_P2065953	Btbd19	2.342753	A_51_P330213	Asf1b	2.2257009
A_55_P2086659	Rad54l	2.3358805	A_55_P2106150	Cenpk	2.2183182
A_52_P75348	Spdl1	2.3287234	A_51_P241645	Tmem194	2.2148042
A_52_P208613	Fancb	2.3260117	A_55_P2000833	E2f8	2.214217
A_55_P2056325	Anxa3	2.3212445	A_66_P128909	Numbl	2.214057
A_51_P405606	Ndrg1	2.316399	A_55_P2122605	Cbr2	2.2092533
A_52_P1026777	Ecscr	2.3122125	A_55_P1994939	Hmgb2	2.1965373
A_52_P155554	Cdc42ep2	2.3115675	A_51_P334942	Aldh1a1	2.1943865
A_51_P237383	Rnase4	2.3097575	A_55_P2002578	Ephx1	2.1877406

A_52_P282838	Spin2	2.3068016	A_55_P2177998	Tmprss13	2.1860015
A_51_P281380	Tspan5	2.3036907	A_52_P231170	Megf9	2.184845
A_55_P2108012	Fam78b	2.3008935	A_51_P260504	Arhgef4	2.1840866
A_55_P2028054	Incenp	2.3003485	A_55_P2016652	Cdk1	2.174548
A_55_P2317665	E2f1	2.2996154	A_52_P158431	Tshz2	2.1675797
A_52_P591166	Dpysl2	2.295929	A_51_P195034	Esco2	2.1546135
A_51_P281089	S100a6	2.2951696	A_55_P1953087	Mcm3	2.1530664
A_51_P191649	Ndc80	2.2937024	A_51_P227392	Rhou	2.1519089
A_55_P2172274	Smc2	2.2912965	A_55_P2020148	Zfp239	2.1510825
A_55_P1968355	Tle1	2.2843323	A_55_P2052290	Psat1	2.1496198
A_55_P2429225	Psrc1	2.2837493	A_51_P469008	Ehd1	2.149087
A_51_P427603	BC018242	2.2758195	A_55_P2133255	Mad2l1	2.1462216
A_51_P351970	Hells	2.2694194	A_51_P231630	Zfp239	2.146201
A_51_P372550	Cgref1	2.26249	A_51_P407248	Antxr1	2.1461911
A_55_P2133165	Wwc1	2.2592406	A_65_P17218	Mndal	2.1428928
A_55_P2062598	E2f7	2.2570715	A_52_P151320	Tnfaip8l1	2.1421633
A_52_P64763	Gen1	2.25424	A_55_P1975021	Rad18	2.1410556
A_52_P556462	Fancd2	2.2481968	A_52_P590535	Fbln2	2.1398883
A_51_P208680	Chtf18	2.136382	A_51_P410918	Tor4a	2.054474
A_55_P2001998	Zfp503	2.1353548	A_55_P1972039	Nox4	2.0544324
A_52_P512817	Shisa5	2.1340823	A_52_P304947	Cenpn	2.0523508
A_55_P2156016	Recql4	2.1329093	A_55_P2000658	Gm15645	2.0498903
A_66_P102090	Pkmyt1	2.1275618	A_66_P104815	Ecm1	2.0469174
A_51_P241791	Mlf1ip	2.1208868	A_52_P98625	Sowahc	2.0467753
A_52_P609024	Cenpw	2.1164157	A_55_P2002129	Rgs12	2.045846
A_55_P2149107	BC055324	2.1159973	A_55_P2024290	Fam149a	2.0442805
A_51_P223709	Pask	2.1151268	A_66_P106113	Rhoj	2.0440416
A_65_P17827	Cenpl	2.1149733	A_55_P2084656	Hist1h2ah	2.0429752
A_55_P2086423	Recql4	2.1061614	A_55_P2039881	5-Sep	2.0421493
A_55_P2085181	Chaf1b	2.1036687	A_51_P356931	Orc1	2.0409997
A_51_P347240	Lrr1	2.101033	A_55_P2042823	Sh3bp2	2.0374854
A_55_P2039320	Zfp365	2.098049	A_55_P2069597	Tspan17	2.036126
A_52_P437795	Cdh11	2.0894513	A_51_P466229	Pdgfrl	2.0351367
A_55_P2032750	Cpt1c	2.0879645	A_55_P2027087	Plcl1	2.0290952

A_55_P2000354	Rgs11	2.0878568	A_51_P190254	Scrn1	2.0285559
A_51_P300709	Srm	2.086297	A_55_P2084652	Hist1h2ak	2.0279403
A_51_P472274	Sox18	2.079722	A_55_P2030536	Ccnyl1	2.0275261
A_55_P2123496	Jam2	2.0759068	A_51_P517051	Gatsl3	2.0267055
A_65_P20433	Parpbp	2.0735905	A_66_P137605	Mb21d1	2.026433
A_55_P2045258	Ehd1	2.0734859	A_52_P106259	Egfr	2.0253048
A_55_P2063465	Thbs3	2.0720322	A_51_P125986	Gan	2.018743
A_51_P427530	Pgm1	2.071374	A_55_P1964787	Slc35e4	2.0096962
A_51_P301394	Gspt2	2.0657253	A_51_P209807	Ccnyl1	2.0070941
A_51_P505823	Endod1	2.061496	A_51_P171288	Gli3	2.005231
A_51_P275976	Dok1	2.0606222	A_55_P2236291	Ppap2a	2.0050724
A_66_P121495	Psat1	2.058627	A_52_P330214	Fanca	2.0049188
A_55_P2138333	Mad2l1	2.0564246	A_52_P436238	Odc1	2.0020258
A_52_P278295	Pear1	2.0552857	A_55_P2457154	G2e3	2.0009356
**Down-regulation**					
**Probe Name**	**Gene Symbol**	**Fold Change**	**Probe Name**	**Gene Symbol**	**Fold Change**
A_51_P363187	Cxcl1	8.819109	A_51_P378051	Aoc3	3.4195278
A_51_P371750	Marco	4.244317	A_51_P509573	Ccl4	3.2854807
A_51_P140710	Ccl3	3.8399758	A_51_P110301	C3	3.143817
A_55_P2011872	Scd4	3.1273491	A_52_P419373	Lrrc39	2.2218037
A_55_P2010066	Capn3	3.066352	A_52_P537827	Wdr72	2.211024
A_52_P638459	Ccl5	3.0502234	A_55_P2131899	Sox4	2.2036295
A_51_P438805	Txnip	2.960222	A_55_P1968958	Cycs	2.1992636
A_55_P1961127	H19	2.7792394	A_55_P2083511	Fam179a	2.1970444
A_51_P485405	Gtsf1	2.753445	A_51_P224064	Klhl38	2.1529965
A_55_P2049717	Agrp	2.614552	A_52_P411430	Gm5914	2.1467266
A_66_P125223	Gm14492	2.5872364	A_55_P2156155	Gadl1	2.1390762
A_55_P2137611	Irgm2	2.507127	A_51_P186476	Slc11a1	2.1028404
A_51_P112355	Igtp	2.500275	A_55_P2012849	Dock10	2.096866
A_55_P2006255	Txnip	2.4396472	A_55_P2144386	Emr1	2.0907123
A_52_P495565	Efnb3	2.4059038	A_55_P2069818	Mapk10	2.0728924
A_55_P2048607	Hp	2.3700798	A_52_P54280	Adck3	2.0677137
A_51_P149714	Ms4a6d	2.3155587	A_55_P2121608	Sox4	2.058894

A_55_P1964598	Hsd17b7	2.2948925	A_66_P119771	Nme8	2.0545104
A_55_P2004213	Gprasp2	2.290954	A_55_P2150757	Gzmm	2.050169
A_51_P134452	Lpar3	2.2610373	A_51_P285413	Rbbp6	2.0317993
A_66_P119968	Clec4a1	2.2528236	A_55_P1984655	Smtnl2	2.0299695
A_55_P2153496	Ppp2r3d	2.241007	A_52_P518808	Mmd	2.0077486
A_51_P126337	Fgf12	2.231632	A_52_P400425	Pirt	2.005233
A_51_P111612	Arrdc4	2.2245975	A_55_P1971174	Cd1d2	2.002264

Table S2. Sequences of cDNAs of FoxM1, Id1, Hmgb2, and Jnk3-shRNAs.

**Selected genes for overexpression in CMs**		
**Gene name**	**Forward 5'-3'**	**Reverse 5'-3'**
FoxM1	CGAAGCGGCCTCACGATGAGAACAAGCCC	CTTGCAGTACTCGCAGCTAAGGGATGAACTG
Id1	CCTGCTCGAGGGATCATGAAGGTCGCCAG	CTCAAGTACTCGCCTCAGCGACACAAGA
Hmgb2	ACGCGAATTCGTCGTCATGGGCAAGGG	CACTGATATCCAGCCACTTATTCTTCATCCTC
**Specific shRNAs targeting Jnk3**		
**shRNAs**	**Targeting sequences**	
Jnk3-shRNA	CAATAGAGAGATCCAACATAA	

Table S3. List of primers utilized in real-time PCR.

Gene name	Forward 5′-3′	Reverse 5′-3′
**Genes from CM reprogramming day 2**		
FoxM1	CACTTGGATTGAGGACCACTT	GTCGTTTCTGCTGTGATTCC
Id1	CGACTACATCAGGGACCTGCA	GAACACATGCCGCCTCGG
Jnk3	AAACTACGTGGAGAATGCGCC	TGGCTTGGCTGGCTTTAAGT
Hmgb2	TGTCCTCGTACGCCTTCTTC	CCTCCTCATCTTCTGGTTCG
E2f8	GAGAAATCCCAGCCGAGTC	CATAAATCCGCCGACGTT
Spon2	CGGCCAAATACAGCATCACC	CCCAGCAGCGAAGACCACT
Plk1	GCCCCTCACAGTCCTCAATA	TACCCAAGGCCGTACTTGTC
Ccl5	TGCGAGGACTCTGAGACAGC	GAGTGGTGTCCGAGCCATA
Fgf12	TGTATCGCCAACAGGAATCAGG	GGAGAGGGAAGAACGGAGAG
**Genes for each functional group in microarray**		
Krt6a	AGAGAGGGGTCGCATGAACT	TCATCTGTTAGACTGTCTGCCTT
Elf3	GGCCCTCATGGCTGCCACCT	TTGGGATCTTGTCTGAGGTCCTGGA
Ocln	ATCCACCTATCACTTCAGA	TAATCTCCCACCATCCTC
Cldn3	GGCGGCTCTGCTCACCTTA	CGTACAACCCAGCTCCCATC
Fgf4	CGTGGTGAGCATCTTCGGAGTGG	CCTTCTTGGTCCGCCCGTTCTTA
Fdps	ATGGAGATGGGCGAGTTCTTC	CCGACCTTTCCCGTCACA
Hmgcs1	GCCGTGAACTGGGTCGAA	GCATATATAGCAATGTCTCCTGCAA
Cgn	CTAAACCGACTTCCTCGATTAA	TGTTGATGAGCGAGTCCACTG
Tjp2	AAGTTCCCTGCCTACGAG	ATTCAACCGAACCACTCC
**Genes for mechanism**		
Cdk4	AGTCAGTGGTGCCAGAGAT	AGATTCGCTTATGTGGGTTA
Cdk2	TGTGCCTCCCCTGGATGAAG	CATCCTGGAAGAAAGGGTGA
Cdk1	GTCCGTCGTAACCTGTTGAG	TGACTATATTTGGATGTCGAAG
p16	GAACTCTTTCGGTCGTACCC	CGAATCTGCACCGTAGTTGA
p21	GCAGATCCACAGCGATATCC	CAACTGCTCACTGTCCACGG
p27	GGGCAGATACGAGTGGCAG	CCTGAGACCCAATTAAAGGCAC
Aurkb	CATCCCTGAGGAGGAAGACC	TTCATAGCAGAGCACCCCG
Mad2L1	TGGTAGTGTTCTCCGTTCGATCT	GCAGGGTGATGCCTTGCT

Table S4. List of *p*-values in each figure.

Figure	*p*-value	Figure	p-value
**Fig 3A**	*p*(Id1)=0.0059<br>*p*(Hmgb2)=0.0214<br>*p*(FoxM1)=0.0245<br>*p*(Jnk3)=0.0096	**Fig 3C**	*p*(F)=0.0349<br>*p*(I)=0.039<br>*p*(H)=0.3225<br>*p*(Ji)=0.0146<br>*p*(FI)=0.0004<br>*p*(IJi)=0.003<br>*p*(FJi)=0.0268<br>*p*(FIJi)=0.0002
**Fig 3D**<br>**Fig 3H**	*p*-value=0.041<br>*p*-value=0.0176	**Fig 3E**	*p*(H3P)=0.0036<br>*p*(AURKB)=0.0123
**Fig 4B**<br>**Fig 4C**	*p*-value=0.0121<br>*p*-value=0.0003	**Fig 4E**<br>**Fig 4F**	*p*-value=0.0008<br>*p*-value=0.0406
**Fig 5C**	*p*(BrdU)=0.0016<br>*p*(H3P)=0.0053	**Fig 5E**	*p*(+dP/dt)=0.0019<br>*p*(-dP/dt)=0.007<br>*p*(ESPVR)=0.0052<br>*p*(PRSW)=0.0247<br>*p*(Tau)=0.0669
**Fig 5D**	*p*(EF%-D1)=0.0743<br>*p*(EF%-D21)=0.0127<br>*p*(FS%-D1)=0.0676<br>*p*(EF%-D21)=0.014	**Fig 5F**	*p*-value=0.0189
**Fig 6A**	*p*(Aurkb-F)=0.0438<br>*p*(Aurkb-I)=0.0331<br>*p*(Aurkb-Js)=0.0059<br>*p*(Aurkb-FIJs)=0.0002<br>*p*(Mad2L1-F)=0.0118<br>*p*(Mad2L1-I)=0.0002<br>*p*(Mad2L1-Js)=0.0055<br>*p*(Mad2L1-FIJs)=0.0091<br>*p*(Plk1-F)=0.0017<br>*p*(Plk1-I)=0.0037<br>*p*(Plk1-Js)=0.0029<br>*p*(Plk1-FIJs)=0.0011	**Fig 6B**	*p*(Cdk4 -I)=0.0196<br>*p*(Cdk4-FIJs)=0.0019<br><br>*p*(Cdk2-F)=0.0344<br>*p*(Cdk2 -Js)=0.0124<br>*p*(Cdk2-FIJs)=0.0074<br><br>*p*(Cdk1-F)=0.0001<br>*p*(Cdk1-I)=0.0334<br>*p*(Cdk1-Js)=0.0124<br>*p*(Cdk1-FIJs)=0.0002
**Fig 6C**	*p*(p16-I)=0.0114<br>*p*(p16-FIJs)=0.0393<br>*p*(p21-F)=0.0002<br>*p*(p21-FIJs)=0.0016<br>*p*(p27-Js)=0.0406<br>*p*(p27-FIJs)=0.011	**Appendix Fig S2B**<br><br>**Appendix Fig S5**	*p*-value=0.0011<br>*p*(F-D4)<0.0001; *p*(F-D21) <0.0001<br>*p*(I-D1)=0.0114<br>*p*(Js-D1)=0.0209<br>*p*(Js-D7)=0.0134<br>*p*(Js-D21)=0.03

Appendix figures

Figure S1

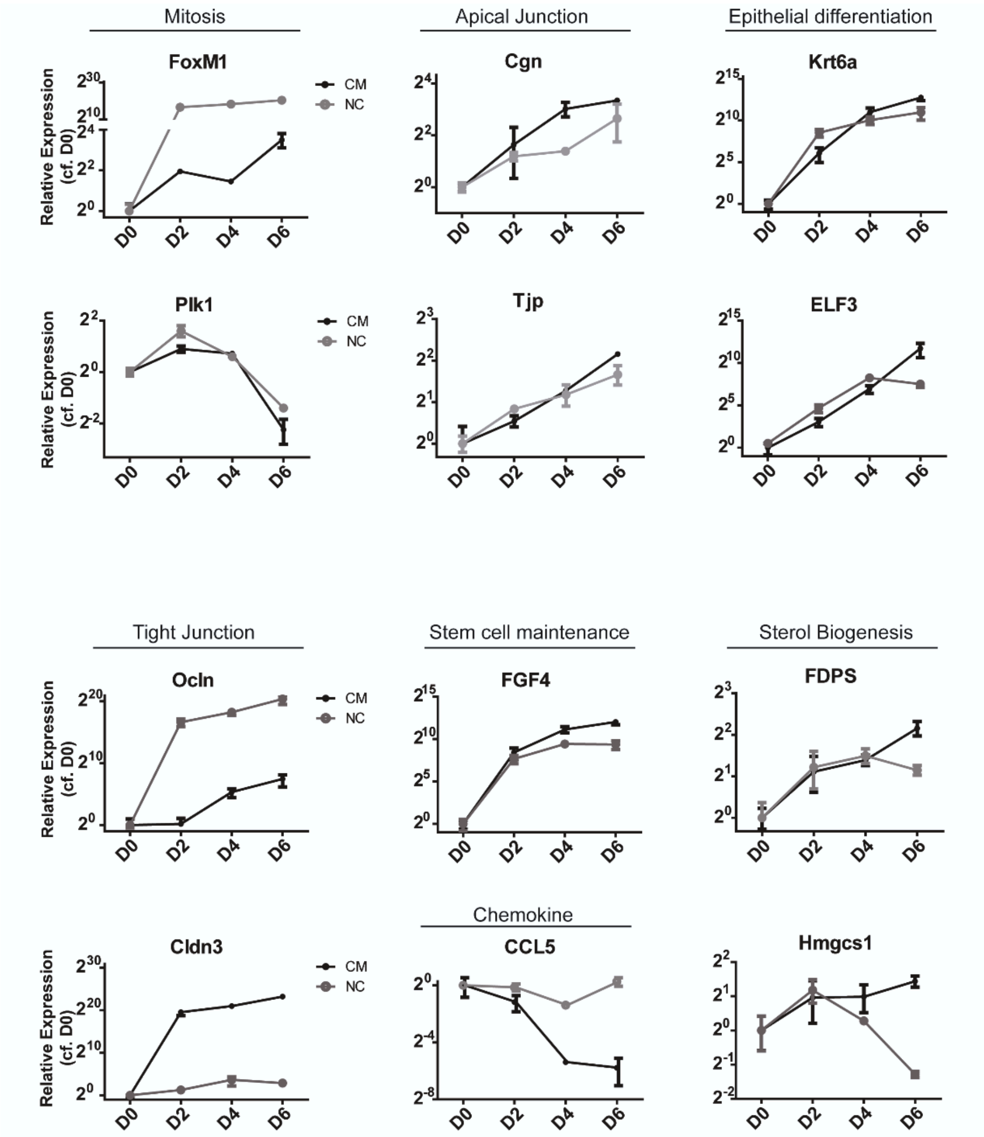

Figure S1. Real-time PCR validation of selected genes in each microarray functional group.

Figure S2

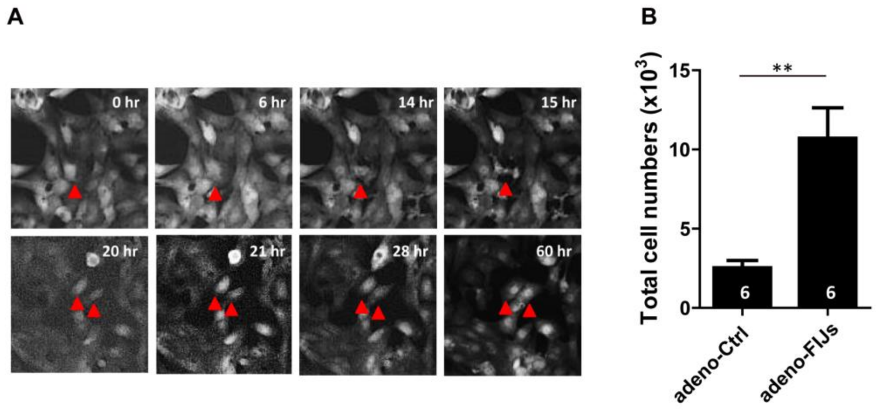

Figure S2. FIJs-treated CMs undergo complete cell cycle to increase total cell numbers

A The time-lapse microscopy of the complete cell cycle of isolated CMs.

B Total numbers of isolated CMs after treating with adeno-Ctrl or adeno-FIJs. **, $P < 0.01$.

Appendix Figure S3

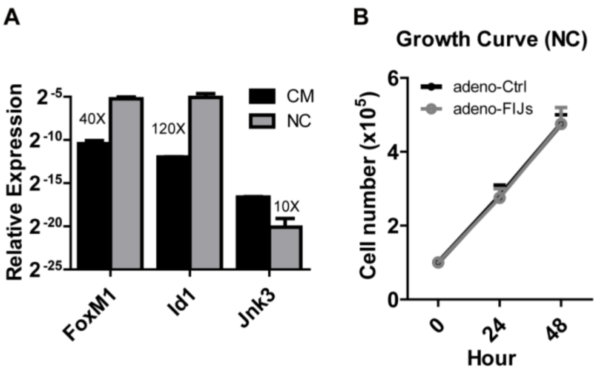

Figure S3. The various effects of the FIJs treatment in the heart.

A RNA expression of cardiac FoxM1, Id1, or Jnk3 in CMs and NCs.

B The growth curve of NCs after adeno-Ctrl or adeno-FIJs treatment.

Appendix Figure S4

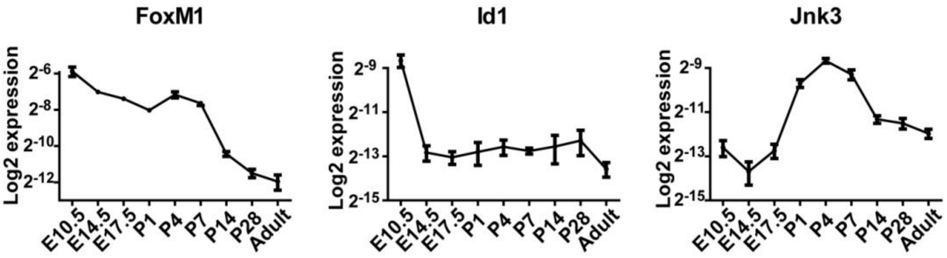

Figure S4. Transcriptional changes of cardiac FoxM1, Id1, or Jnk3 during development.

Appendix Figure S5

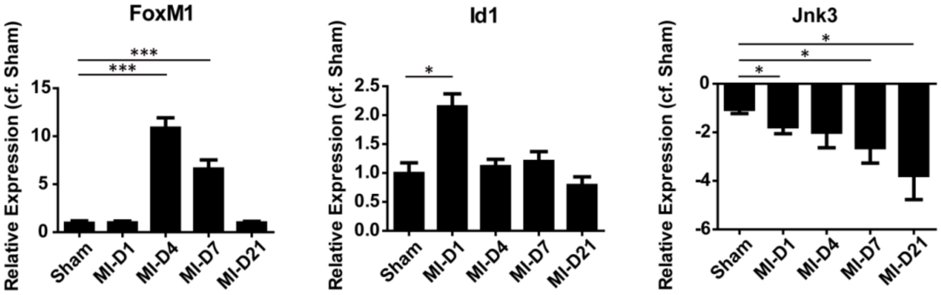

Figure S5. Transcriptional changes of cardiac FoxM1, Id1, or Jnk3 after MI.

*, $P < 0.05$, and ***, $P < 0.001$.

Appendix Figure S6

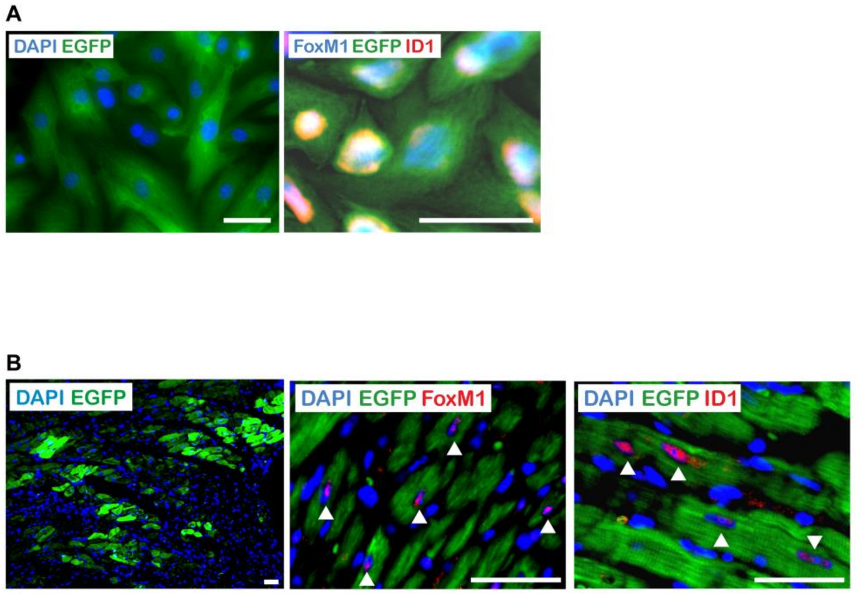

Fig S6 Adenoviral infection efficiency of CMs after adeno-FIJs treatment.

A EGFP expression combined with immunofluorescence staining of FoxM1 and Id1 in CMs after adeno-FIJs treatment *in vitro*.

B Immunofluorescence staining of EGFP and FoxM1 or Id1 in CMs after adeno-FIJs treatment *in vivo*.

The scale bar represents 50 µm.
